# Label-Free Imaging and Histo-Optical Evaluation of Head and Neck Cancers with Multiphoton Autofluorescence Microscopy

**DOI:** 10.3390/cancers15041302

**Published:** 2023-02-18

**Authors:** Paula Patricia Villarreal, Rahul Pal, Suimin Qiu, Orly Coblens, Alejandro Villasante-Tezanos, Vicente Resto, Susan McCammon, Gracie Vargas

**Affiliations:** 1 The Institute for Translational Sciences, University of Texas Medical Branch, Galveston, TX 77555, USA; 2 Athinoula A. Martinos Center for Biomedical Imaging, Massachusetts General Hospital, Charlestown, MA 02129, USA; 3 Department of Pathology, Division of Surgical Pathology, University of Texas Medical Branch, Galveston, TX 77555, USA; 4 Department of Otolaryngology, Head & Neck Surgery, University of Texas Medical Branch, Galveston, TX 77555, USA; 5 Department of Biostatistics and Data Science, School for Public and Population Health, University of Texas Medical Branch, Galveston, TX 77555, USA; 6 Department of Otolaryngology, Head & Neck Surgery Oncology Division, The University of Alabama at Birmingham, Birmingham, AL 35294, USA; 7 Department of Neurobiology, University of Texas Medical Branch, Galveston, TX 77555, USA

**Keywords:** multiphoton microscopy, autofluorescence, second harmonic generation, squamous cell carcinoma, head and neck, epithelium, label-free imaging

## Abstract

**Simple Summary:**

Efforts to identify lesions of head and neck cancers with high malignant potential are important to improve patient outcomes, as 5-year survival rates remain low due to late detection. Optical imaging approaches that provide direct cellular and structural atypia could be helpful in detection and pathology guidance. This study evaluates the method of multiphoton autofluorescence microscopy (MPAM) for its ability to reveal atypia associated with neoplasia in resected tumor samples without the need for exogenous dyes or tissue sectioning, showing a correlation with the corresponding histology.

**Abstract:**

Depth-resolved label-free optical imaging by the method of multiphoton autofluorescence microscopy (MPAM) may offer new ways to examine cellular and extracellular atypia associated with epithelial squamous cell carcinoma (SCC). MPAM was evaluated for its ability to identify cellular and microstructural atypia in head and neck tissues from resected discarded tumor tissue. Three-dimensional image volumes were obtained from tissues from the floor of the mouth, tongue, and larynx, and were then processed for histology. MPAM micrographs were evaluated for qualitative metrics of cell atypia and quantitative measures associated with nuclear pleomorphism. Statistical analyses correlated MPAM endpoints with histological grade from each imaged site. Cellular overcrowding, discohesion, anisonucleosis, and multinucleated cells, as observed through MPAM, were found to be statistically associated with dysplasia and SCC grading, but not in histologically benign regions. A quantitative measure of the coefficient of variance in nuclear size in SCC and dysplasia was statistically elevated above histologically benign regions. MPAM also allowed for the identification of cellular heterogeneity across transitional areas and other features, such as inflammatory infiltrates. In the future, MPAM could be evaluated for the non-invasive detection of neoplasia, possibly as an adjunct to traditional conventional examination and biopsy.

## 1. Introduction

There are 1.1 million head and neck cancer diagnoses annually, making it the seventh most common cancer worldwide [1]. The recent SARS-CoV-2 pandemic may have led to a rise in head and neck squamous cell carcinoma (HNSCC) cases as individuals are foregoing clinical care [2]. Common malignancies in the head and neck SCC include cancers in the oral cavity, such as the floor of the mouth (FOM), tongue, and the pharynx or larynx, the latter being a difficult site to access for screening [3]. Head and neck SCC is associated with notable morbidity because of the functional importance of the structures affecting speech and eating. Furthermore, survival is dependent on the stage of the disease [4]. The 5-year survival rate at advanced stages with distant metastases for FOM is 20% and 36% for tongue carcinoma [5]. However, the 5-year survival rate can increase to 75% and 78%, respectively, when cancers at these sites are localized and diagnosed at the primary region. This survival rate can potentially increase if eradicated prior to invasion [5]. Thus, efforts to identify neoplasia at early stages, including detection of lesions with high malignant potential, are important to improve the outcomes [4,6,7,8,9,10].

Currently, HNSCC screening is performed through conventional oral examinations (COEs), which require the visual recognition of high-risk lesions by a skilled practitioner [11]. The final diagnosis is based on histological identification of well-defined atypia based on cellular and epithelial changes, including morphometry, which is indicative of abnormal proliferation and differentiation [12]. Though most identified precursor lesions are not malignant, it is critical that those with a high risk of transformation as well as early cancers are detected. COE often fails to discriminate between low-risk lesions and lesions of high malignant risk [11,13,14]. Therefore, tissue assessment methods that provide an indication of cellular atypia could be a powerful bridge between COE and diagnosis based on histology.

Optical imaging techniques have been investigated for potentially bridging this gap as they offer the ability to evaluate microstructural changes in intact tissues and are amenable to miniaturization for clinical imaging [15,16,17,18,19,20]. Multiphoton microscopy, which provides subsurface visualization of cellular and subcellular architecture based on fluorescence, which may be performed in a label-free manner without dyes or stains, could potentially provide this bridge between traditional screening and pathology for improved diagnostics [21,22]. This rapidly growing technique utilizes near-infrared light to penetrate deeper into tissues and obtain depth images of microarchitecture, and has been widely used to study and understand cellular and molecular features of health and disease [23,24,25,26,27]. When applied without the use of dyes or stains, termed multiphoton autofluorescence microscopy (MPAM), the sources of contrast are endogenous fluorophores, such as flavins and extracellular matrix components. A contrast often integrated in multiphoton microscopy is second harmonic generation microscopy (SHGM), which provides non-invasive imaging of fibrillar collagen due to its specificity for non-centrosymmetric molecules. MPAM and SHGM are highly complementary and may be co-registered to delineate the complex microstructure of the epithelium in two-and three-dimensional views. The ability of MPAM and/or SHGM to define all cellular atypia and collagen architecture in oral dysplasia and SCC has been demonstrated in animal models [7,8,9,10,24,26,27]. However, applications of the technology have been studied on a limited basis in human head and neck tissues, primarily to demonstrate the feasibility of imaging by multiphoton microscopy for cell features and microstructure, but no studies have performed a quantification or statistical correlations to evaluate image metrics against histology [28]. It is noted that a different but related method of higher harmonic generation imaging in human oral cancer tissue showed the ability to image cell and collagen organization, but also did not quantitatively define the image metrics for statistical comparison against histology [29]. Multiphoton microscopy technology has been developed for clinical use in select dermatological applications, but with no designated clinical systems for head and neck cancers, which is likely because the imaging instruments were too large for the oral cavity [30,31]. However, several developments in fiber-based instrumentation have led to research grade multiphoton microscope borescopes/probes, making future clinical translations a possibility [32,33,34,35].

Though MPAM shows promise for delineating cellular and extracellular atypia associated with neoplasia, the lack of quantitative studies in human head and neck cancers serves as a motivation for the current study. The goal of this study was to perform a histo-optical MPAM assessment of resected head and neck tumors in order to provide an indication of agreement with the gold standard of histology. This study serves as an assessment for the potential clinical translation of this modality to detect dysplasia and SCC in humans. A label-free MPAM was performed on resected patient tumors from three different anatomical sites: the tongue, FOM, and the larynx. Histological features used in identification of dysplasia and SCC, such as multinucleation of cells, cellular discohesion, and cellular overcrowding, were visualized and analyzed using MPAM across benign, dysplastic, and SCC sites obtained from resected samples. A quantitative cell morphometry parameter, nuclear coefficient of variance (CoVa), a feature that has been shown to differentiate between benign/inflammation, and neoplasia in animal model studies of oral neoplasia, are also assessed [8]. Results in the current study indicate that MPAM provides a valuable histo-optical assessment for the detection of atypia in dysplasia and SCC, which indicates that this method may be promising for potential clinical translation.

## 2. Materials and Methods

### 2.1. Clinical Sample Collection

Oral cancer tissues from 23 patients who have had surgical resections to remove primary tumors of the head and neck (8 tongue, 10 FOM, and 5 larynx) were used in this study. This tissue was obtained under a discarded tumor tissue protocol approved by the University of Texas Medical Branch Institutional Review Board. Deidentified discarded tissues were provided by pathology following examination by a head and neck pathologist. Samples were immediately placed in Dulbecco’s Modified Eagle Medium (DMEM) without phenol red.

### 2.2. Imaging System

Multiphoton autofluorescence microscopy was performed using an upright multiphoton excitation fluorescence microscope (Ultima IV, Bruker, Middleton, WI, USA) employing a Mai Tai femtosecond laser (Spectra Physics, Mountain view, CA, USA) for fluorescence excitation. The excitation wavelength was centered at 800 nm. Fluorescence emission was obtained over a broad emission band (480 to 650 nm) using GaAsP photomultiplier tubes for detection (Hamamatsu, Japan). Microscopy was performed using a 40 × 0.8 N.A. water immersion objective with a working distance of 3 mm (MRD07420, Nikon), providing an imaging field of view of 321 × 321 µm. A z-interval of 1 µm was used in z-stack acquisitions. Collection of second harmonic generation for imaging of fibrillar collagen, when obtained, was accomplished using a narrow bandpass filter at 420 nm with the illumination laser set at 840 nm. Imaging was performed within 2 h of obtaining the samples from pathology and time to scan was 10–15 min per excitation wavelength. Prior to MPAM, a gross white light image was taken of the specimen. Samples were mounted on a 30 mm cage plate (CP06, ThorLabs, Newton, NJ, USA) with phenol-free DMEM media. The sealed sample holders had a #1.5 cover glass and the mounted samples were placed on the microscope motorized stage with the mucosal side facing the cover glass for *en face* imaging. For each clinical sample provided, multiple, separated sites were imaged and a punch biopsy was used to obtain the imaged site from the main specimen. Between 4–6 imaged sites, with corresponding biopsies, were obtained for each clinical sample by MPAM/SHGM, resulting in a total of 130 regions obtained from 23 patient specimens (30 imaged sites were from tongue, 67 imaged sites were from FOM tissue, and 33 imaged sites were from laryngeal tissue). Two additional patient samples that were too narrow to image *en face* were not included in analysis, but were imaged in cross-section for features that MPAM may reveal in this orientation (shown in Supplementary Materials, Figure S1).

### 2.3. Histology Preparation and Pathological Evaluation of H&E

Acquired biopsy tissues for each imaged site were immediately placed in 10% formalin for twenty-four hours and submitted for histological processing. Samples were embedded in paraffin, sectioned, and stained with hematoxylin and eosin (H&E) for histological examination by a head and neck pathologist. Histopathology grading was given for each site in accordance with World Health Organization criteria [36], with sites categorized as benign, oral epithelial dysplasia (OED), or squamous cell carcinoma (SCC). Grading of H&E histology was conducted in a blinded manner, with samples performed *en masse*. These gradings served as ground truth for imaged sites in statistical analysis.

### 2.4. Image Feature Analysis

Cellular morphological qualitative and quantitative analysis was performed on image stacks. First, qualitative gradings associated with cell and nuclear changes traditionally used in histopathology to assess epithelial dysplasia and SCC were evaluated. These comprised several categorical variables indicating absence or presence (0 = no, 1 = yes, >= 25% of the volumetric stack) of cellular atypia: Anisonucleosis, cellular discohesion, overcrowding of cells, and cellular multinucleation, graded through volumetric image stacks acquired for each site. Anisonucleosis in MPAM micrographs was defined as autofluorescent cells with abnormal variation in nuclear size and shape. Overcrowding, as visualized through MPAM, was recognized as presence of increase in localized, cluster-like nuclei overlapping one another. Cellular discohesion appeared as fields showed loosened intercellular connections between squamous cells. Sites with cells with multiple nuclei were categorized as multinucleated. A single grader, trained on training sets from preclinical oral dysplasia, cancer tissues, and clinical tumor samples not included in this study, performed final scoring while blind to histological outcome.

For quantitative analysis, CoVa of nuclear area (nuclear CoVa), defined as the mean of the nuclear area divided by the standard deviated of the nuclear area, was measured in the mid-layer (~60 to 80 µm in depth) of the volumetric stacks [8]. In each stack, three separate image planes that were ~10 µm apart were analyzed by measuring 20 nuclei from each plane. Using the Fiji Image J software, calibration of the individual stacks was set to 3.18 µm/pixel, in accordance to the objective lens FOV and sampling [37]. The “length tool” in the FIJI software was used to measure the lengths of the major and minor axes of each nucleus by manually extending a line across nuclei along the major and minor axes, measuring the length in microns. The shape of nuclei of the cells imaged were ellipsoidal, thus the area of each nucleus (~60 nuclei from each image stack) was estimated using the general formula for area of an ellipse given as the following:

(1) Area of a Nuclei=π∗Length of Major Axis∗Length of Minor Axis 

Analysis was performed by a single researcher (PV) who was blind to pathological grade at time of measurement and analysis.

### 2.5. Statistical Analysis

#### 2.5.1. Categorical Histomorphometric Analysis

For statistical analysis of the qualitative, categorical variables, a contingency table was created and the percent probability of a histomorphometric feature observed as acquired using MPAM was calculated as follows:

(2) $$Probability\;\left(\%\right)=\frac{Histomorphometric\;feature\;}{\#\;total\;sites}\times 100$$

Chi-square test of independence was used to analyze presence or absence of association between categorical variables and histopathology grading. A correlogram, a visual representation of associations, was then constructed to show the degree of association between MPAM qualitative grading and histopathology grading [36]. The correlogram shows an increased association with positive residual value (blue gradient) and a decrease in association with negative residual values (red gradient) from the Chi-square test using residual analysis. This analysis identifies which specific cells in the contingency table are producing the greatest contribution to the Chi-square test result by standardizing the distances between the observed and expected responses, a measure of goodness of fit.

#### 2.5.2. Analysis of Nuclear CoVa

For evaluation of the quantitative continuous parameter of nuclear CoVa, a histogram was made and Shapiro–Wilk’s test was performed to check normality. A Kruskal–Wallis test (non-parametric approach to the one-way ANOVA) was performed to compare the three diagnostic groups by the dependent variable of nuclear CoVa. Subsequent Dunn’s test, a non-parametric pairwise analysis alternative to post hoc, was carried out to test multiple pairwise comparisons for significance between the median values of each tested group.

#### 2.5.3. Multinomial Regression of Continuous and Categorical Data

To encompass the categorical and continuous data into a single analysis, a multinomial regression model was developed, with histopathological grading as an outcome, to elucidate a predictive model of what MPAM image features are associated with neoplasia. Specifically, a baseline category logit model, a type of multinomial logistic model, was performed to give a summary of the odds of an outcome (benign, dysplasia, or SCC) in one category relative to the others. The benign classification was treated as the reference level for comparison with SCC or dysplasia. Subsequently, dysplasia was compared against SCC. In all statistical tests, alpha was set at 0.05 and analyses were performed using R software version 4.0.2. The R software libraries in this study for statistical analysis are as follows: multinomial regression-MASS, odds ratio-epitools, and multicollinearity: corrplot.

## 3. Results

### 3.1. MPAM/SHG Tissue Imaging

**Figure 1:**
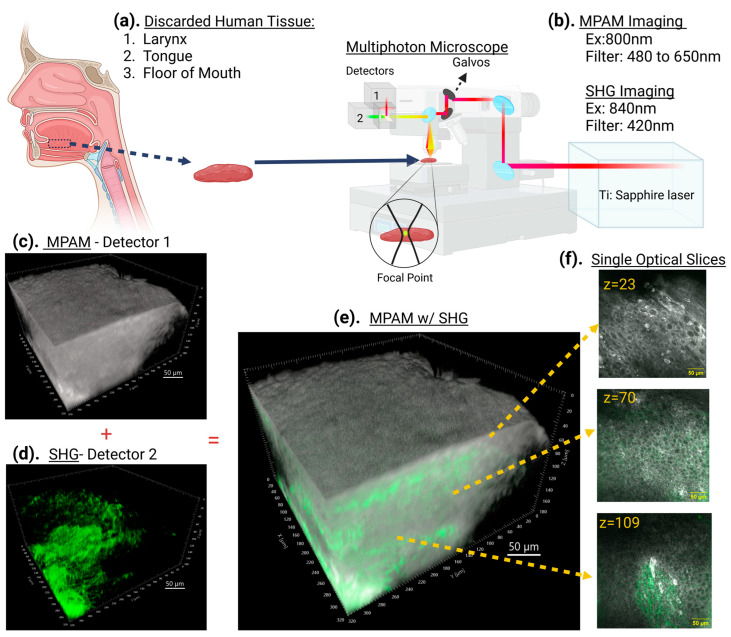
Schematic of MPAM and SHG imaging of resected head and neck human tumors. Multiphoton microscopy uses short femtosecond pulses of near infrared light to produce fluorescence in the visible region of the light spectrum in a laser scanning microscope configuration. Fluorescence excitation and, thus, emission are restricting to the objective focal point and scanning the laser in three-dimensions results in a 3D microscopy. When applied to unstained tissue, the source of fluorescence contrast is from intrinsic fluorophores in tissues. The workflow for imaging in this study was as follows: (**a**) discarded human tissue from tumors obtained from the larynx, tongue, or FOM were (**b**,**c**) imaged using 800 nm excitation for MPAM (pseudo colored in gray) and (**d**) 840 nm excitation for SHG from collagen (pseudo colored in green). The combination of these two z-stacks (**e**) provides a volumetric view of a region of interest on the collected sample. Optical sectioning of single slices at different depths (**f**) provides detailed observation at each depth, such as in these micrographs showing epithelial cells (round structures with a dark nucleus and bright autofluorescent cytosol) and collagen (green). Scale bar: 50 μm.

The MPAM/SHG imaging workflow (Figure 1) enabled a volumetric view of the regions of interest from the intact collected sample. An advantage of this technique is the ability to further analyze the optical slices that make up the volume for more detailed views of the epithelial cells and surrounding collagen at different depths.

### 3.2. Label-Free MPAM/SHG Depth-Resolved 3D Imaging of Head and Neck SCC Tissue

**Figure 2:**
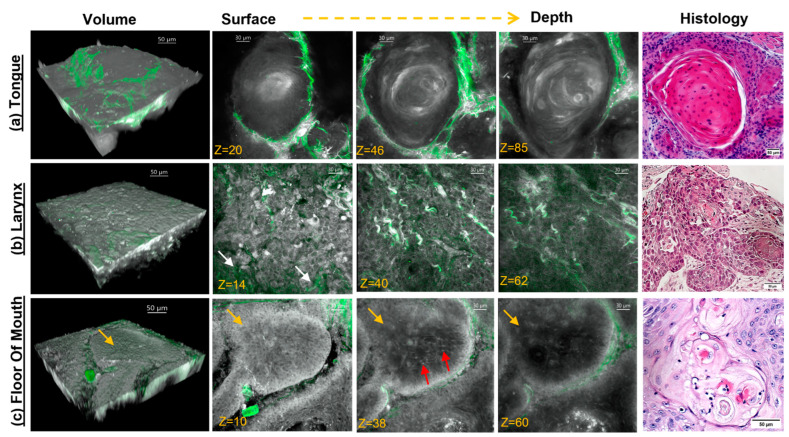
Volumetric and single plane MPM-SHGM images of SCC in human tongue (**a**), larynx (**b**), and floor of mouth (**c**). First panel represents a 3D reconstruction with MPAM autofluorescence in gray and SHGM from fibrillar collagen in green. Depth panels (z indicates image slice) display superficial, intermediate, and basal epithelial layers with corresponding histology shown in rightmost panel. (**a**) In tongue, a keratin pearl is seen with concentric keratin rings and surrounded by collagen (green). (**b**) A typical laryngeal tumor had superficial thick collagen bundles (identified by white arrow) interspersed among squamous epithelium, with epithelial cells identified by a bright (autofluorescent) cytosol and dark nucleus. (**c**) Floor of mouth also had similar epithelial structure. Here, a squamous island is identified by yellow arrows and surrounding collagen can be seen in green. Bright puncta (identified by red arrows) show possible inflammatory infiltrates. Scale bar in volumes and histology: 50 μm. Scale bar in depth micrographs: 30 μm.

Figure 2 demonstrates typical volumetric and depth-resolved MPAM/SHG images of the human head and neck tumors from the tongue, larynx, and floor of the mouth with corresponding H&E. Figure 2a shows the representative, volumetric, and single-depth planes in tongue SCC, with MPAM shown in gray and SHG in green. In this example, a keratin pearl is identified by the appearance of concentric keratin rings. Fibrillar collagen SHG, shown in green, surrounds the keratin pearl. The features of this ringed pattern strongly resemble that of a keratin pearl of the corresponding H&E micrograph in the rightmost panel. In laryngeal tumors (Figure 2b), individual epithelial cells can be identified clearly in single planes as having a bright cytosol with dark central nuclei. Collagen bundles were found near the surface of laryngeal tumors, with bundles interspersed between the epithelial cells near the surface (indicated by white arrows). Epithelial cell and extracellular matrix organization in MPAM micrographs is comparable to the corresponding H&E. In FOM (Figure 2c), a squamous island of cells (noted as yellow arrows) can be seen with the surrounding fibrillar (green) collagen, both in the first volumetric image and in the individual depth images. This feature was consistent with the corresponding histology. Occasional bright puncta in MPAM are likely infiltrating inflammatory cells (noted by red arrows), scattered throughout the squamous island, such as seen in the H&E. Although small amounts of collagen are seen in this example surrounding the squamous island, it was not consistently found in all cases. Separately, Figure S1 shows an example of a provided tissue sample in a cross-section taken from the tongue and showing a regular epithelial organization with a typical distribution of collagen shown by SHG in green.

### 3.3. Qualitative Identification of Cellular Features Associated with Dysplasia and SCC

Some key features of cellular atypia commonly used in histological grading in diagnosing OED and SCCs are cellular overcrowding, cellular discohesion, multinucleation of individual cells, and anisonucleosis/pleomorphism. Examples of these cellular features identified in MPAM imaged stacks are shown in Table 1. For each histological grading, the percentage of samples displaying the given cellular atypia are shown. Because several atypia features can occur in a single imaged z-stack, the sum of the percentages will not equal 100%. Cellular overcrowding, defined as a cluster of cells overlapping, can be seen in the representative micrograph (Table 1). This event was observed in 6% of benign regions, while dysplasia showed an 85% occurrence, and SCC an 97% occurrence. Loosened intercellular connections between squamous cells are recognized as cellular discohesion (noted by yellow arrow), which occurred in 16% of benign sites versus 45% in dysplastic areas and 80% in SCC regions. Polynuclear squamous cells, or multinucleated cells, were observed in 16% of the benign sites compared to 85% in dysplastic regions and 93% of the SCC sites. Anisonucleosis (abnormal variation in nuclear size) and nuclear pleomorphism (an abnormal variation in nuclear size and shape), were qualitatively identified in 6% of benign sites. The percentage increased to 90% for dysplastic regions and was noted 100% of the time in SCC areas.

**Figure 3:**
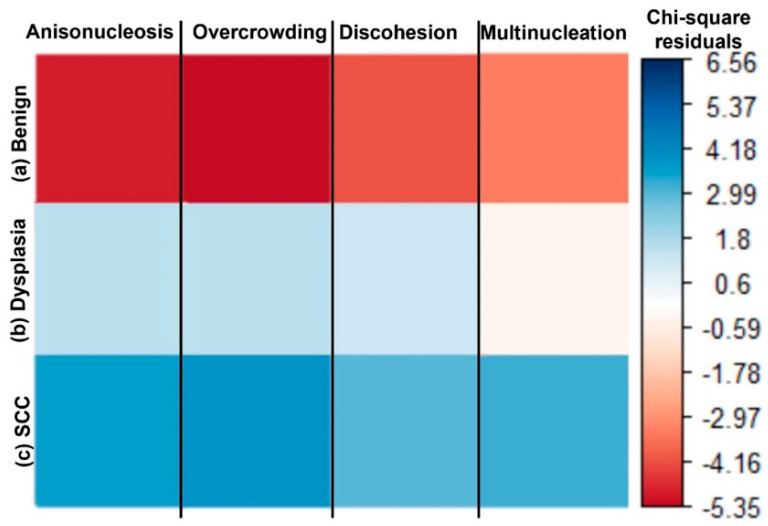
A correlogram developed from Chi-square residuals shows the degree of association between histological gradings and atypic cellular features scored in MPAM image stacks. An increased association with positive residual value is shown in blue and a decrease in association with negative residual values from the Chi-square test is shown in red. Benign sites (**a**) were negatively associated with each atypic feature. Dysplastic sites (**b**) had a positive association with all atypic features, except for the case of multinucleated sites. SCC sites (**c**) displayed a strong, positive association with all atypic features.

The contingency table for Table 1 was used to assess if there were significant associations between histological gradings versus each category of atypic cellular feature by the Chi-square test, regardless of anatomical site. Respective Chi-square scores (X^2^), degrees of freedom (df), and *p*-values per cellular criteria are as follows: (1) anisonucleosis-X^2^ = 110.18, df = 2, and *p*-value < 2.2 × 10^−16^, (2) overcrowding-X^2^ = 111.53, df = 2, and *p*-value < 2.2 × 10^−16^, (3) discohesion-X^2^ = 44.847, df = 2, and *p*-value = 1.826 × 10^−10^, and (4) multinucleation-X^2^ = 74.261, df = 2, and *p*-value < 2.2 × 10^−16^. The *p*-values less than 0.05 indicate a significant association between each MPAM cellular feature and histological grading. A correlogram showing the residuals from the Chi-square scores is shown in Figure 3. SCC sites had a positive association (blue) with anisonucleosis, cellular discohesion, overcrowding, and multinucleation, while the benign sites were negatively associated (red). Sites graded as dysplastic had a positive association (blue) with all features but the multinucleated cells (red hue). These results indicate a statistical association between atypic features and neoplasia.

Finally, though this comprised a limited number of samples, we calculated the odds ratios (ORs) of each histological grading against the presence or absence of each cellular feature, to examine the potential for the increased odds of finding cellular atypia according to the histological grade (Table 2). In this preliminary analysis, the benign sites had a consistent OR of one, indicating that the odds do not change in displaying an atypia feature by MPAM. The benign sites also served as a reference group for OR comparison. Dysplastic sites, however, all have an OR greater than one, indicating higher odds of having atypia compared to benign sites. The odds in the case of SCC were greater than that of dysplasia or benign, indicating atypic cellular features are more likely to occur in SCC. In some instances, the confidence intervals (CI) show a wide range of values, which is an indication of sparse data. Nonetheless, these show a trend toward a high statistical significance of MPAM scores for each histological grading.

### 3.4. Nuclear CoVa, a Continuous Measure of Anisonucleosis, Significantly Increases in Sites of Dysplasia and SCC Relative to the Benign Sites

**Figure 4:**
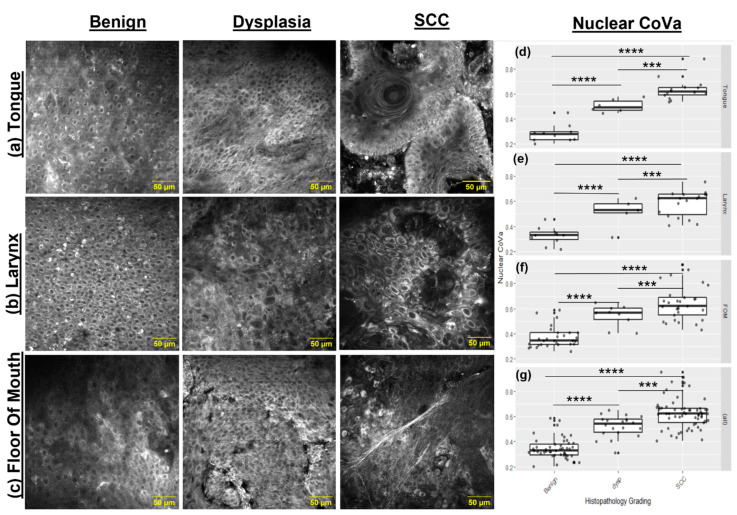
Representative, single mid-layer micrographs of (**a**) tongue, (**b**) larynx, and (**c**) floor of mouth and respective pathological grading (benign, dysplasia, and SCC). Benign regions displayed an organized cellular structure. Dysplastic regions showed some nuclear atypia, with some variable nuclear shape and size. SCC presented the most variation in nuclear shape, as noted in panels (**d**–**f**). Panel (**g**) shows all grouped diagnostic samples, regardless of anatomical site. Scale bar, 50μm for optical slices, Kruskal–Wallis test with subsequent Dunn’s test, *p*-value < 0.0001 = ****, and *p*-value < 0.001 = ***. Dunn’s test, *p* value.

Epithelial organizations for benign, dysplastic, and SCC sites in the tongue, larynx, and floor of the mouth along with CoVa measures are shown in Figure 4. Analysis of nuclear CoVa from MPAM indicated an increase in value with grade determined by histopathology for the anatomical tissues of the tongue, larynx, and the floor of the mouth. In benign sites, the regularity of the nuclear size can be appreciated in Figure 4a. The nuclear CoVa mean values were typically 0.3, with a low deviation, indicating a fairly uniform nuclear area throughout the sites and consistency across the anatomical regions. The dysplastic sites showed a mixture of nuclear atypia (varying nuclear size and shape) with organized nuclear morphometry similar to benign cases, which in some cases, was evident within the same field of view. Nuclear CoVa in dysplastic cases was consistently higher than that of benign, with a typical mean value of 0.5. In every case, SCC presented the most variation in the nuclear area, although it is typically larger than that found in dysplastic and benign sites, as noted in the box plots in Figure 4d–f. Figure 4g shows data compiled from all anatomical sites, categorized by histopathological grade, and shows a consistent trend to Figure 4d–f. A statistical analysis by Kruskal–Wallis test showed a *p*-value of less than 0.05, indicating a significant difference in at least one pair of the diagnostic groups. Dunn’s test revealed the significance across all possible pairwise comparisons in the groups categorized by histopathological grading. A multinomial multivariable analysis between categorical variables and the continuous variable of nuclear CoVa is shown in Supplementary Materials, Table S1.

### 3.5. Transition Areas and Additional Features Identified by MPAM

**Figure 5:**
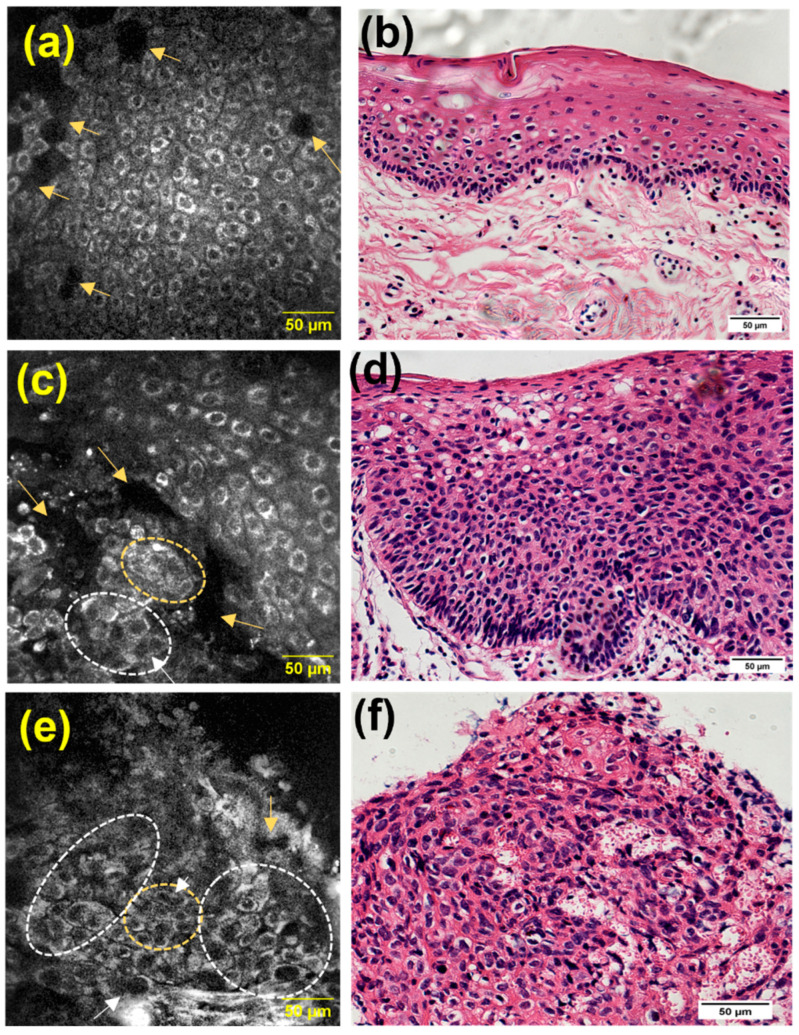
MPAM visualization of an intact FOM tissue. An organized, uniform cellular pattern can be seen in panel (**a**) and is also evident in corresponding histology (**b**). In a neighboring site graded as a dysplastic site (**c**,**d**), cytologic atypia and cellular discohesions are visible. Full fields of cellular abnormalities were found in an adjacent area to that of the dysplastic site (**e**,**f**). For example, cellular discohesion (indicated by yellow arrow) and anisonucleosis (indicated by the white circle) can be seen in all gradings. Overcrowding (indicated by yellow circle) and multinucleated cells (indicated by white arrow) can be observed in the dysplastic and SCC graded sites. Microscopy FOV 321 × 321 µm. Histology was imaged with Obj. 20×, 0.4 NA Scale: 50 µm.

To demonstrate the potential of MPAM to capture variations across tissues with transitional areas, MPAM imaging was performed across individual intact samples. Figure 5 shows the intact sample of an FOM tissue, in which the heterogenous cellular morphology can be seen across the three regions. In the first sampled area of Figure 5a, a uniform pattern of epithelial squamous cells with similar size and shape is seen with corresponding H&E in Figure 5b. This organization was consistent with the benign tissue structure. Some cellular atypia, such as cellular discohesion (indicated by yellow arrow), can be observed in this benign example. The overall regular cellular organization is gradually lost across the transition, as shown by MPAM (Figure 5c,e) and the corresponding histology in Figure 5d,f. Specifically in Figure 5c,d, cytologic abnormalities such as nuclear atypia/anisonucleosis (indicated by white circle), multinucleated cells (shown as white arrows), overcrowding (highlighted in yellow circle), and discohesion (denoted by yellow arrow) can be observed in this site graded as dysplasia. This cellular atypia is further enhanced in Figure 5e,f, which shows full fields of all cytologic abnormalities evaluated in this study.

**Figure 6:**
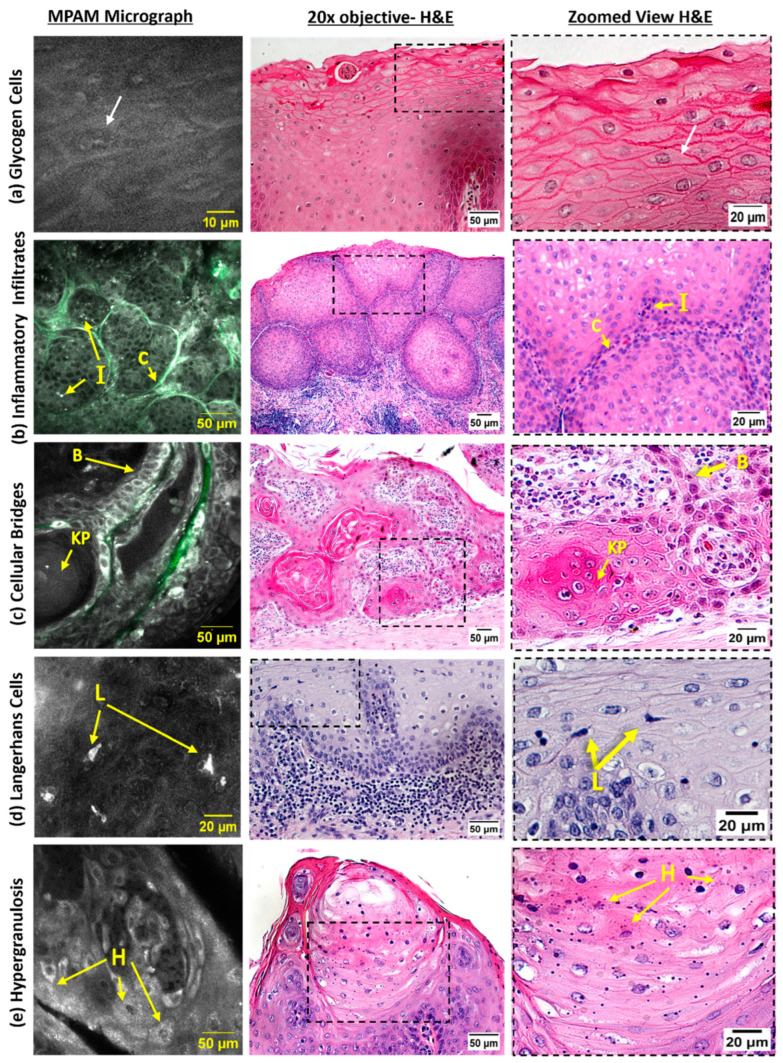
Additional features identified with MPAM in intact specimens from head and neck tumors. MPAM images from tumors (first column) with corresponding H&E following imaging. (**a**) Glycogenic cells (identified by white arrows) are seen in the first few layers of the epithelium. Immune infiltrates (**b**), noted as “I”, can be found interspersed in squamous islands, surrounded by collagen (shown in green and identified by “C”). Cellular bridges (**c**), noted as “B”, can be seen connecting to a keratin pearl (“KP”), with strands of collagen (colored green) nearby. Langerhans cells (**d**), emphasized by “L”, can be found in the epithelial mid-layers of a region of interest. Hypergranulosis (**e**), identified as “H”, can be observed near the surface of the tissue. Microscopy FOV 321 × 321 µm.

A variety of additional features and cell types (e.g., infiltrating inflammatory cells) that have been investigated for aiding diagnostics when seen in histology were visible by MPAM in some cases and are shown in Figure 6. Figure 6a shows MPAM panels consistent with the morphology of glycogenic cells with a bright delineated cell membrane with dark cell body and a bright perinuclear/nuclear space (noted by a white arrow). Figure 6b shows inflammatory infiltrates observed as small bright lobular-shaped puncta and, at times, with visible multinucleation (denoted by “I”). Such infiltrates were located in the epithelial islands surrounded by collagen (marked as “C”). Figure 6c indicates the presence of cellular bridges (symbolized as “B”), as small, lined squamous cells that connect to other squamous epithelial islands. In the case shown, a keratin pearl (denoted as “KP”) can be seen to be connecting directly to the cellular bridge. Figure 6d shows an autofluorescent pyramidal cell (marked as “L”) with a consistent characteristic morphology of Langerhans cells. Figure 6e shows an example of hypergranulosis (noted as “H”), with indications of chronic irritation o, the surface of the tissue.

## 4. Discussion

Despite being in accessible sites of the head and neck, SCCs are most often detected at the late stage of disease beyond localized regions, indicating the need for additional tools for early detection of early cancers and high-risk dysplasia. Our motivation for this study was to evaluate the MPAM imaging technique as a label-free method for histo-optical visualization and as an evaluation of head and neck neoplasia, specifically to detect cellular and microstructural atypia associated with neoplasia, which could be explored for future clinical use. This was conducted with imaging regions of resected tumor specimens (Figure 1) from the FOM, tongue, and larynx, which were then evaluated against H&E histology from those specific regions. The novelty of this work lies in the application of this method in human oral tissue, being the first to quantitatively and statistically evaluate MPAM image metrics for cell atypia against histology. This work provides a glimpse of what this method may offer if translated into use in a clinical setting for ex vivo pathology assessment or even in vivo application. The focus at this time was to determine the potential to detect atypia against the gold standard of histology rather than evaluate for patient-based diagnosis.

MPAM provided *en face* depth-resolved volumetric images of resected tissues, with image planes revealing features consistent with cellular atypia found in neoplasia (OED or SCC) that similarly resembled the cellular morphology of corresponding same-site H&E histology (Figure 2). SHG microscopy enabled the visualization of collagen (Figure 2 and Figure S1), which is an important feature to explore as it is known to undergo progressive remodeling and degradation in neoplasia [8,23,24,38]. The cellular features imaged by MPAM for the qualitative assessment of atypia (Table 1) were chosen to parallel those commonly used in diagnosis using histology. Chi-square and residual statistical analyses of MPAM visual features indicated that atypic cellular features acquired through MPAM were positively associated SCC gradings and negatively associated with benign gradings (Figure 3). Dysplasia had a positive association (blue) with all features but multinucleated cells, otherwise trending similarly to SCC. The subsequent odds ratio analysis (Table 2) provided an indication for the increased odds of finding cellular atypia in SCC and dysplasia, though it should be considered with caution due to low sample size, which was noted in the wide range of confidence intervals. In the future, a larger sample analysis could be repeated to confirm the increased odds of MPAM atypic features in dysplasia and SCC.

The quantitative measure of nuclear CoVa was motivated by previous in vivo MPAM studies in a hamster preclinical model, in which this continuous variable was found to discriminate between benign/inflammatory and dysplastic conditions [8,39]. In fact, we found a significant increase in the nuclear CoVa in head and neck dysplasia and SCC sites compared to sites graded as benign by the histological assessment (Figure 4). This held true for all three anatomical locations evaluated (tongue, FOM, and larynx) and indicates that nuclear CoVa may be a promising image-based marker to potentially discriminate dysplasia and SCC from benign sites in head and neck tissues. This measurement of nuclear CoVa was made possible by the contrast provided by the cytosol surrounding the nucleus. In the future, it would be of interest to directly evaluate signals from the endogenous metabolic fluorophores of the cytosol such as, NADH and FAD, which are known to play a critical role in the early changes in epithelial cancers [39,40], which were preliminarily explored *ex vivo* in head and neck tumor samples [28].

While not statistically evaluated, an additional advantage that MPAM may confer is the intact assessment of tissue to capture possible heterogeneity of cellular morphology (Figure 5) and other cell types, as shown in Figure 6. Observations in micrographs included inflammatory infiltrates, glycogenic cells, cellular bridges, Langerhans cells, keratin pearls, and instances of hypergranulosis. Associations between oral SCC and chronic inflammation have been found in each of these cell types [41,42]. Specifically, Langerhans cells have been reported in oral squamous cell carcinoma, though they decreased in OED [43], and hypergranulosis has been described in hyperkeratosis cases, which is a potential early sign of premalignant lesions [44], a feature that could be leveraged in the development of pathology detection and diagnosis strategies.

The approach of per site comparison with histology provided an indication that features identified by imaging corresponded with that of the histology findings, both in the qualitative feature scores and the quantitative nuclear feature measures. Thus, while our assessment does not per se test the potential for patient diagnosis, it provides a critical step in the development of approaches that could help in high-risk lesion and SCC detection as well as diagnosis. One possible role could be to provide histo-optical metrics of atypia at the time of the pathological assessment. Indeed, this application of MPAM has been explored in some diseases [45,46]. Such an application could be carried out with a benchtop MPAM instrument. Another possibility for development could be as a tool providing biopsy guidance, necessitating the development into an in vivo imaging tool such as a borescope or endoscope. Currently, there are portable CE-certified class 1 M clinical multiphoton tomograph systems for dermatological applications that have undergone safety studies; however, they are too bulky for oral cavity assessment [30,47]. Studies of multiphoton systems such as those used clinically have indicated that the safety with photodamage was reported to be comparable to exposure to UV radiation [47,48,49]. In the current study, comparable powers to CE-certified clinical tomograph systems were used with the same laser parameters but at a lower NA (0.8 vs. 1.3), indicating the use of lower fluences than those used in the existing dermatological systems. The miniaturization of fibered, handheld multiphoton instruments that are compact enough to reach anatomical structures in the oral cavity has recently been developed in laboratories [32,33,34,35]. Therefore, clinical implementation of miniaturized multiphoton systems may occur in the near future. Studies, such as the current investigation, provide critical steps toward evaluating the future clinical potentials for point-of-care detection and adjunctive diagnosis.

In comparison with other microscopy modalities investigated in head and neck SCC, namely, confocal microscopy and optical coherence tomography (OCT), MPAM imaging in this study provided a contrast based on autofluorescence with imaging to several cell layers below the surface [50]. OCT provides morphometry based on reflected light and has been shown to resolve layers of the in vivo human buccal mucosa and may provide value in scattering-based image features for dysplasia detection [19]. This technique, however, has a significantly lower spatial resolution than MPAM. Confocal methods offer subcellular resolution imaging similar to MPAM. In ex vivo SCC human tissue sections, confocal fluorescence microscopy has shown cellular pleomorphism, anisocytosis, and destruction of basal membrane, but it required an exogenous dye [51,52]. Confocal reflectance microscopy translated into clinical systems may aid in the detection of atypic nuclear density in skin and oral epithelium based on reflected light, but it is still being evaluated for its full potential for head and neck SCC [15,18,53,54,55,56]. Confocal endoscopy with fluorescence as a contrast mechanism has also been applied for head and neck SCC investigations and has shown promise; however, it requires the use of exogenous contrast agents, which are generally non-specific with the few FDA approved agents available [17,57,58]. In terms of the source of contrast, a potential advantage of MPAM over confocal imaging is the ability for label-free imaging without the need for exogenous contrast agents and the potential for simultaneous collagen imaging as well as signatures from intrinsically metabolic fluorophores, as discussed above. MPAM also typically provides considerably more in-depth penetration, which is dependent upon tissue and image parameters, rather than the confocal microscopy [59]. However, studies that make specific comparisons between modalities are needed to evaluate the capabilities and limitations of each.

One limitation of this study was the limited sample size, thus serving as a feasibility study. With larger samples, more comprehensive ORs and multinomial models can potentially serve for predictive diagnosis. A preliminary multinomial logistic regression merging categorical and continuous variables to develop a statistical model for detecting benign, dysplastic, and SCC consistent sites indicated that MPAM may be helpful in detecting atypia (Table S1). The relatively small sample size limited this multinomial regression model, which is indicated by large odds values and wide range of CIs. A broader study with additional samples and one in which investigations are performed specifically to test ability for diagnosis will need to be performed to fully evaluate the potential power of this imaging method. Of note, collagen was not always observed in SCC tissues due to the thickness caused from proliferative cells. With greater sample sizes, the chances of observing collagen would increase and metrics with statistical impact pertaining to collagen density/surface area would be of interest to explore. Patient demographic information was also not collected, as the samples were obtained through a deidentified and discarded tissue IRB. This limited imaging to sites on discarded tumor samples requires that respective histology from image sites be used for comparisons of the image data. However, the results of these site-based comparisons indicate that MPAM helps identify atypic cellular features and shows feasibility for detection that can be explored in future assessments to include non-tumor tissues from control subjects.

## 5. Conclusions

Abnormal cellular architecture serves as an important indicator for tissue grading and the diagnosis of lesions with high malignant potential and cancer in traditional pathology. Here, we present the use of label-free MPAM for the histo-optical assessment of sites on resected tumor samples, without the need for tissue sectioning or exogenous labeling. This study is the first to show quantitative and statistical associations between cellular atypia parameters evaluated by MPAM and the histological grade in clinical head and neck tumor samples, establishing it as a promising method for future use in early detection efforts, such as a component of the pathology workflow or a point-of-care imaging tool.

## Figures and Tables

**Table 1:** Histopathological cellular features commonly used for histological grading can be visualized using MPAM. Representative whole and zoomed-in micrographs from tumors are shown next to feature criteria. Cellular discohesion is highlighted by yellow arrow. A tabulation of the percentage of all samples displaying the defined feature are shown for each histopathological grading (benign, dysplasia, and SCC) for all tumors imaged. Scale bar: 50 μm for full micrograph view; 10 μm for zoomed-in micrographs.

Cellular Atypia Identified by MPAM in Resected Tissue					
Features	Representative MPAM Micrographs		Histopathological Grading		
	Full View	Zoom-in View	Benign	Dysplasia	Squamous Cell Carcinoma
Overcrowding: Increase of localized, cluster-like nuclei overlapping	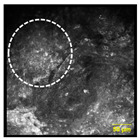	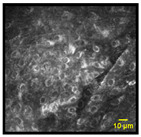	6%	85%	97%
Cell Discohesion: loosened intercellular connections between squamous cells	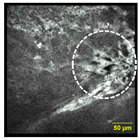	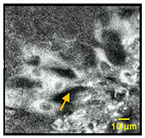	16%	45%	80%
Multinucleated Cells: Squamous cells that appear poly nuclear or have more than one nuclei per cell	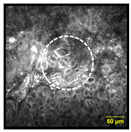	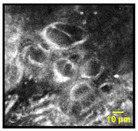	16%	85%	93%
Anisonucleosis/Nuclear Pleomorphism: Abnormal variation in nuclear size and shape	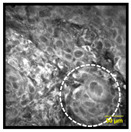	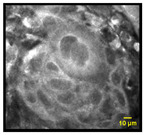	6%	90%	100%

Note. Total 23 Patients, n = 130 Sites.

**Table 2:** Odds ratios (ORs), a measure of association between the histopathological grading and the assessed variables, were calculated in each instance. Benign grading was used as a reference group. Sites graded as dysplastic had increased odds compared to benign in every instance. SCC had the greatest odds ratios compared to dysplasia and benign. It is noted that this is a sparse dataset with confidence intervals showing a wide range of values.

Odds Ratios Increase as Histological Grade Severity Increases			
Measured Variable	Benign	Dysplasia	SCC
	OR *	OR CI	OR CI
Anisonucleosis	1	69 (17–544)	702 (82–32,417)
Overcrowding	1	208 (24–10,071)	964 (110–50,236)
Discohesion	1	3 (1.3–13)	17 (7–51)
Multinucleation	1	19 (6–96)	52 (19–209)

Note. OR = Odds Ratio, CI = Confidence Intervals, * = Reference Group.

## Data Availability

No further datasets are available. Existing data frames are available upon request.

## Supplementary Materials

The following supporting information can be downloaded at: https://www.mdpi.com/article/10.3390/cancers15041302/s1. Figure S1: Example MPAM-SHGM view of a thin tongue sample imaged in cross-section. Table S1: Preliminary statistical analysis of the final baseline logit multinomial model developed from quantitative and qualitative measurements based on MPAM acquisitions.
