# Supplementary material for: Label-Free Imaging and Histo-Optical Evaluation of Head and Neck Cancers with Multiphoton Autofluorescence Microscopy
Source: Cancers (Basel). 2023 Feb 18;15(4):1302. doi: 10.3390/cancers15041302 (PMC9953923; doi:10.3390/cancers15041302)
Supplement: Supplementary file 1 [file cancers-15-01302-s001.zip › cancers-2114823-supplementary.pdf]

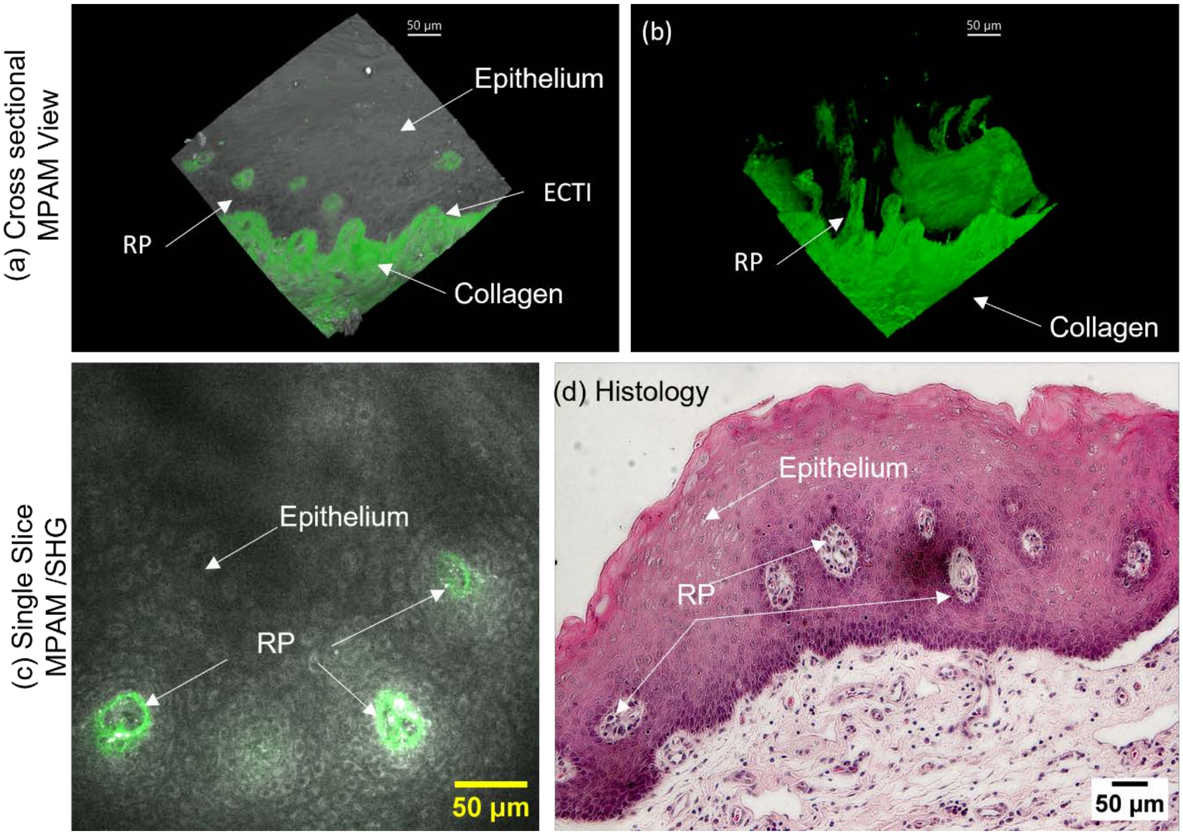

Figure S1. Example MPAM-SHGM view of a thin tongue sample imaged in cross-section (**a**) The sample which was too thin in the lateral direction to imaging *en face* was imaged in cross-section shown here as a volumetric MPAM-SHGM projection, gray representing MPAM arising largely from epithelial cells and green representing SHG from fibrillar collagen, seen both as round areas around rete pegs (RP) and defining the epithelial connective tissue interface (ECTI). (**b**) same volume showing only SHG component of fibrillar collagen (**c**) Single slice MPAM (gray) and SHGM (green) showing epithelial cell organization in gray with fibrillar collagen of rete pegs in green. The columnar, rete pegs of the *lamina propia* are observed as round structures in this tangential cut including the corresponding H&E in (**d**). This tissue was not included in analysis as imaging orientation differed from the analyzed samples. Microscopy FOV 320x320 µm. Scale bar 50µm.

Supplementary Text and Table S1: A preliminary multinomial multivariable analysis indicates MPAM measures of nuclear CoVa, cellular overcrowding, and cellular discohesion could be possible predictors for benign, dysplastic, and SCC grading.

Based on the data analyzed above, we can assume there are significant associations between the analyzed MPAM qualitative (chi-squared) and quantitative (ANOVA- Kruskal Wallace) measures with respective histological gradings. In order to perform multivariate analysis to assess what combination of cellular features, as imaged by MPAM, are most predictive of histological grade, a multinomial logistic regression model was developed. Because anisonucleosis is a measure of nuclear CoVa, there is collinearity between them and, thus, anisonucleosis was excluded from the model. Each variable was first evaluated univariately, and if significance was shown, the variable was added to the model. This model, termed a saturated model, was then simplified through use of the Akaike information criterion (AIC), likelihood ratio tests (LRT), and residual deviances, tests performed for model simplification without impacting performance. The final model was chosen to fit adequately while following the rule of parsimony.

The final multinomial model included only the following variables: nuclear CoVa, overcrowding, and cellular discohesion. The exponentiated odds and confidence intervals of each comparison paired with its p-value are shown in **Table S1**. An odds ratio was calculated for each categorical variable (overcrowding and cellular discohesion) in each comparison pair. An odds greater than one indicates that atypia features are more likely to be present relative to baseline comparison (shown as the denominator of the first column), while controlling for all other variables. For example, in the last grouping (SCC/dysplasia) one can see there is a 4.54 increase in odds that sites graded as SCC have cellular discohesion in comparison to dysplastic sites, with the lower CI of 1.32 and the highest CI at 15.76. For the continuous variable of nuclear CoVa in a multinomial regression, a positive beta coefficient indicates a positive increase the estimated odds of one category relative to the baseline reference (denominator in comparison pairs). For example, in the first grouping of dysplasia vs. benign, the estimated odds that a site was dysplastic is 3.5e+5 times the estimated odds that the site is benign, controlling for the rest of the predictors. Most corresponding odds ratios and confidence intervals demonstrated in this model have a wide range of values, an indication of sparse data. Though preliminary, this data shows such a multivariable model could be a promising diagnostic predictive model using cellular features as captured through MPAM.

Table S1. Preliminary statistical analysis of the final baseline logit multinomial model developed from quantitative and qualitative measurements based on MPAM acquisitions. Categorical variables (cellular discohesion and overcrowding) which are based on the logorithmic odds per 1 unit change should be exponentiated to reflect the odds ratio. Continuous variables (nuclear CoVa) are not based on logarithmic odds and are represented by the estimate only without an odds ratio.

Final Multinomial Logistic Regression Model

Comparison Pairs	Measured Variables	Estimate	Odds Ratio	95%CI		*p*
				Lower CI	Upper CI	
Dysplasia Benign	Nuclear CoVa	12.79	-	1.19	24.39	*0.03
	Cellular Discohesion	1.01	2.74	0.15	50.55	0.49
	Overcrowding	12.36	2.6e+6	2.5e-45	2.6e+55	0.83
OSCC Benign	Nuclear CoVa	22.94	-	10.02	35.85	***<0.0001
	Cellular Discohesion	2.52	12.45	0.62	250.38	0.09
	Overcrowding	12.69	3.2e+5	3.2e-45	3.3e+55	0.82
OSCC Dysplasia	Nuclear CoVa	10.14	-	3.08	17.19	*0.004
	Cellular Discohesion	1.51	4.54	1.32	15.76	*0.01
	Overcrowding	0.24	1.27	0.15	10.47	0.82

Note. No Missing Data, n=130 sites

p-value= 0.05 *

p-value=0.0001 ***
